# A quality audit of MRI knee exams with the implementation of a novel 2‐point DIXON sequence

**DOI:** 10.1002/jmrs.350

**Published:** 2019-07-29

**Authors:** Matthew Bastian‐Jordan, Sanjay Dhupelia, Morgan McMeniman, Matthew Lanham, Jacqueline Hislop‐Jambrich

**Affiliations:** ^1^ QLD X‐ray QEII Hospital, Coopers Plains Brisbane Queensland Australia; ^2^ University of Queensland Brisbane Queensland Australia; ^3^ QLD X‐ray Greenslopes Private Hospital Brisbane Queensland Australia; ^4^ QLD X‐ray Upper Mount Gravatt Brisbane Queensland Australia; ^5^ Canon Medical ANZ North Ryde New South Wales Australia

**Keywords:** DIXON, fat suppression, knee, MRI, soft tissue resolution

## Abstract

**Introduction:**

The objective of this study was to evaluate the effect on diagnostic image quality and acquisition time utilising a DIXON sequence to replace two standard proton density (PD) fat saturation (FS) sequences in routine magnetic resonance (MR) evaluation of the knee.

**Methods:**

Thirty‐one consecutive patients referred for an MR examination of the knee were examined using the routine departmental protocol along with the addition of a DIXON sequence. The sequences were all evaluated by a senior radiologist and feedback provided via both written and scored responses. The sequences were then repackaged for two additional reviewers with the sagittal PD FS (Chemical Shift Selective Fat Saturation or CHESS) and sagittal PD removed and replaced with the DIXON (fat suppressed and in‐phase, respectively) sequence equivalents. Scored and written responses were tabled and reviewed to assess the suitability of sequence replacement.

**Results:**

The DIXON‐based images were judged as being comparable replacements for the sagittal PD fat sat and PD sequences. There was no report of any loss in diagnostic confidence across the 31 patients (total of 32 knees) with a time saving of just over 10% gained. The most common issues raised affecting image quality, though not affecting diagnostic attributes, were patient motion and a minor chemical shift artefact.

**Conclusion:**

The use of the DIXON technique in place of the PD sequences was of equivalent diagnostic quality with’good’ to ‘outstanding’ fat suppression observed for the majority of cases using the DIXON sequence with an incremental time saving obtained.

## Introduction

MRI has long been the imaging modality of choice to assess intra‐articular pathology due to its inherent soft tissue resolution. The utility of this imaging modality however means that the clinical demand is great; therefore, the shorter the time spent on the scanner for a diagnostic series, the more efficient the imaging process overall. During our most recent institutional review of current sequence use across all examination types, our vendor provided us a pre‐release version of their 2‐point DIXON sequence. We performed an audit review of this sequence to assess any impacts on either study time and/or image quality.

The DIXON method has been used clinically for some time to achieve fat suppression via the exploitation of the different processional frequencies of fat and water protons.^1^ This chemical‐shift based method has the added advantage of providing fat only and water only images, as well as in‐phase and out‐of‐phase image sequences from the one acquisition. Variations in this method have more recently led to the development of methods that can be routinely used in fast spin echo (FSE) musculoskeletal applications.^2^, ^3^

The original 2‐point DIXON method is a chemical shift proton‐imaging technique using the in‐phase and out‐of‐phase cycling of fat and water resonance. Novel 2‐point methods however involve the use of in‐phase and partially‐opposed‐phase (POP) images to iteratively estimate the *B*_0_ field map which is used to calculate water and fat images.^4^ These novel sequences have been shown to deliver shorter acquisition time and superior fat saturation when compared to conventional clinical sequences.^5^

With the growth in both the number and the type of MR procedures performed clinically, it is of the greatest importance to minimise the time spent on the scanner such that waiting times are minimised and patient access is maintained. Knee imaging is a common clinical request across most MRI centres, and access to sequences that can be used to save time while maintaining or improving diagnostic utility is therefore vital.

## Methods

Our single centre study was conducted as a quality audit, and therefore, the need for institutional ethics was waived by Metro South Human Research Ethics Committee. We prospectively recruited all adult patients referred for a routine knee MRI (i.e. non‐contrast) over a 2‐month period. The patients were identified from the adult MRI referrals that presented to the institution. Thirty‐one patients were scanned between November 2017 and January 2018. The patients were referred for various clinical issues mainly involving pain, trauma or mobility impairment. As this was an audit we did not have any exclusion criteria, thus patient inclusion was continuous until our quota for the study was reached. One patient was imaged using the 16 channel NeoSoft flex coil due to his high BMI (35.8 kg/m) and cross‐sectional knee size, and all others were scanned using the routine 16 channel QED knee coil. There were 20 males and 11 females (one female had a bilateral study counted as two knees for the purposes of the audit). One patient also had a metallic implant, and no changes were made to the sequence series for this patient. The age range of the patients was 14–78 years (mean 44.6 +/− 16.6), and the body mass index BMI ranged 21.2–39.4 kg/m^2^ (mean 28.12 +/− 5.2). The DIXON sequence was run first in sequence order for all patients.

The MR examination was conducted using a commercially available scanner operating at 3T (Titan 3T V3.4 Toshiba Medical Systems Tochigi, Japan). The novel 2‐point DIXON sequence used was made available to the institution as a pre‐release sequence as part of a research agreement. A total of five sequences were conducted (4 institutional routine sequences, and the DIXON) with technical factors as listed in Table 1. A sagittal proton density (PD) fat saturated (FS) (i.e. DIXON water) and a sagittal PD (i.e. in‐phase DIXON) image series were reconstructed after scanning and included for review.

**Table 1:** Acquisition parameters.

Parameter	PD Sagittal	PD Sagittal FS	PD Sagittal dixon	PD Axial FS	PD Coronal
PE FOV (cm)	14	14	14	14	14
RO FOV (cm)	14	14	14	14	14
Matrix PE	320	320	320	320	288
Matrix RO	416	416	416	368	384
Resolution (PE)	0.43	0.43	0.43	0.43	0.48
Resolution (RO)	0.33	0.33	0.33	0.38	0.36
Phase wrap	1.4	2	2	1.4	1.6
TR	2809	2958	3445	2700	3095
TE	28.5	27	33	27	28.5
Slices	24	24	24	24	24
Thickness (mm)	3.5	3.5	3.5	3.2	3.2
Gap (mm)	0.5	0.5	0.5	1.2	1.0
Speeder PE	1.6	1.2	2	1.8	1.2
NAQ	1	1	1	1	1
ETL	11	11	11	9	11
ScanTime (min.sec)	1.30	2.40	3.41	1.43	2.04
Flip angle	160	160	160	140	180

ETL, echo train length; NAQ, number of acquisition; PE, phase encoding; RO, read out; TE, echo time; TR, repetition time.

Reviewer 1 (MBJ – 6 years of experience) was provided all sequences and made comment on image quality, as well as the suitability to use the DIXON sequence as a replacement for the routine FS sagittal sequences. Direct comparisons between the sequences was performed and scored. Reviewers 2 and 3, both MSK‐trained radiologists (MM with 6 years of experience and SD who has 7 years of experience), were provided with only the two unchanged routine series (axial and coronal PD FS) as well as the DIXON replacement reconstructions, that is a total of four sequences. Reviewers 2 and 3 were asked comparable image quality questions to reviewer 1, without any access to the two routine sagittal sequences that had been replaced.

The analysis used a questionnaire format comprising of Likert scales asking about image quality (1: outstanding, 2: good, 3: some quality issues, 4: poor) (Table 2) with the data analysed with the Spearman Correlation (Table 3). Images were viewed on the local PACS.

**Table 2:**
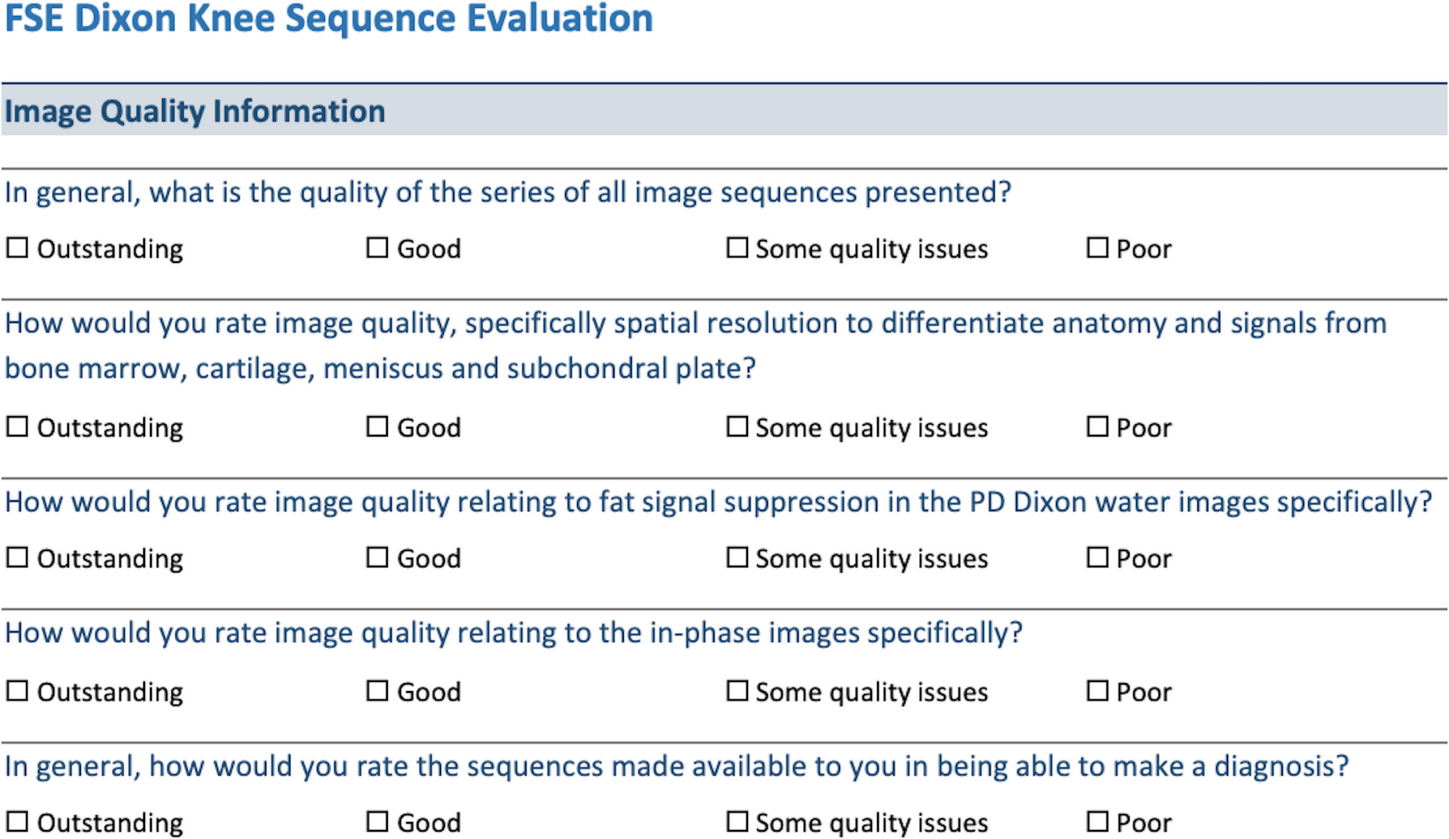
Image quality questions.

1, outstanding; 2, good; 3, some quality issues; 4, poor.

**Table 3:** Image quality answers.

	*N*	Min	Max	Sum	Mean	Std. Dev.
Image quality rating	32	1	3	50	1.56	0.669
Anatomy differentiation	32	1	3	45	1.41	0.615
Fat suppression image quality	32	1	3	38	1.19	0.471
In‐phase image quality	32	1	3	50	1.56	0.716
Diagnostic attributes	32	1	3	48	1.50	0.672
Image quality rating	32	1	3	66	2.06	0.354
Anatomy differentiation	32	2	3	69	2.16	0.369
Fat suppression image quality	32	1	3	64	2.00	0.254
In‐phase image quality	32	1	3	65	2.03	0.309
Diagnostic attributes	32	1	3	67	2.09	0.390
Image quality rating	32	1	3	60	1.87	0.492
Anatomy differentiation	32	1	3	65	2.03	0.309
Fat suppression image quality	32	1	3	60	1.87	0.554
In‐phase image quality	32	1	3	67	2.09	0.390
Diagnostic attributes	32	1	3	66	2.06	0.354

1, outstanding; 2, good; 3, some quality issues; 4, poor.

Colours: reader 1 (pink), reader 2 (green) and reader 3 (blue).

## Results

Reviewer 1 scored all images between 1 and 2 (good to outstanding) for all components with an overall image quality rating of 1.56 and a fat suppression rating of 1.19 (Table 3). The fat suppression was compared against the reviewers’ usual experience in MRI and also between the sequences. There was no loss in quality of fat saturation using a DIXON method, and this was actually improved in the one patient with a metallic implant. In‐Phase image quality was directly compared against the PD FSE sequences with an average score of 1.56 (SD = 0.716) demonstrating good to excellent image quality. Reviewers 2 and 3 (who did not have the standard non‐DIXON sagittal images to compare) also demonstrated scores averaging 1.5–2 and were comparable in their assessment of image quality, fat suppression and diagnostic quality. The ability to identify anatomical structures including cartilage, ligaments, menisci and the subchondral plate was graded as good to outstanding and comparable with the non‐DIXON imaging.

**Figure 1:**
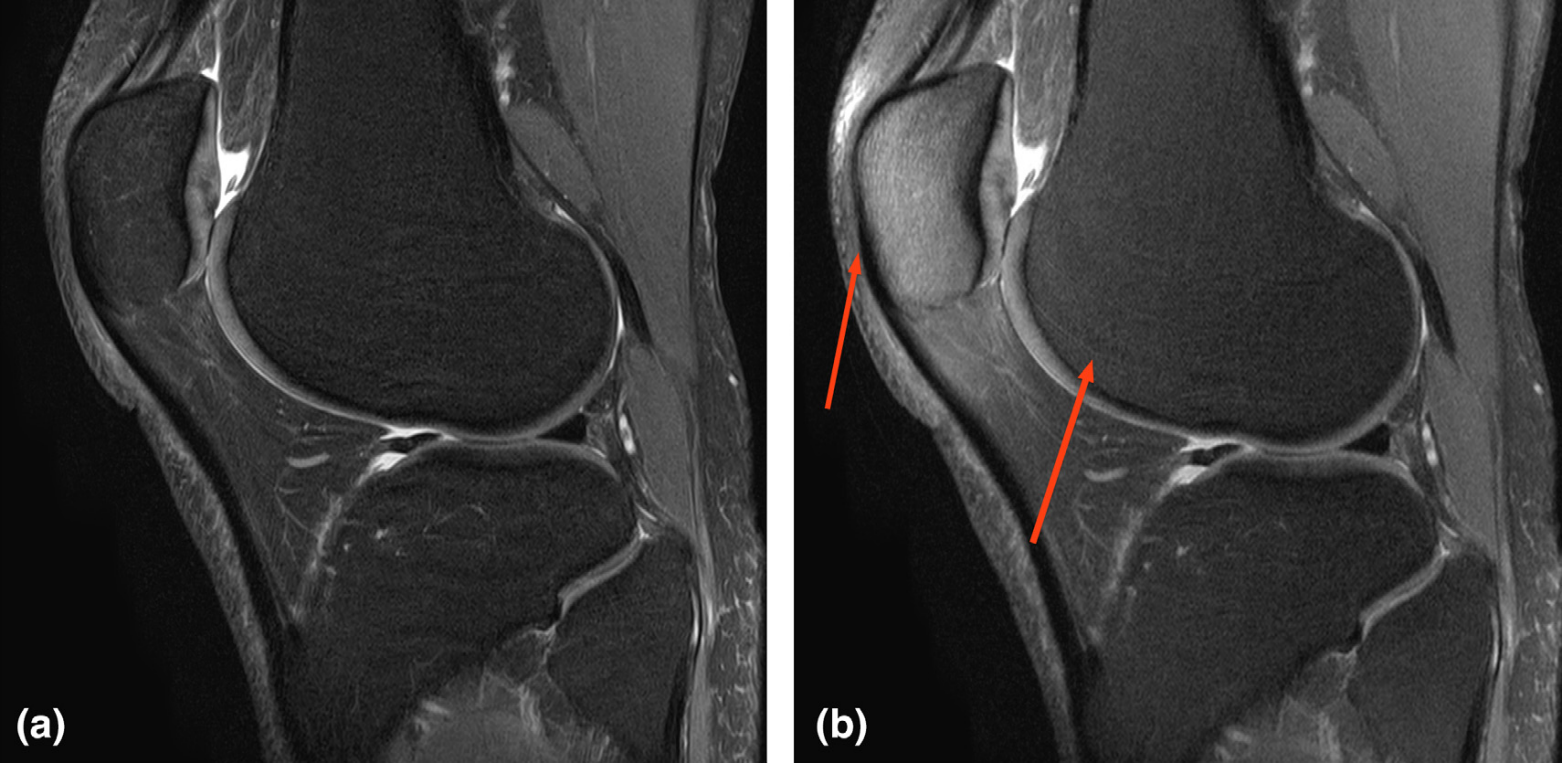
Movement: Image (a) is the DIXON image and b the standard. Note that both images are minimally affected by movement, the higher SNR and superior fat saturation on the DIXON is evident especially around the patella.

Patient motion was mentioned by the reviewers as affecting image quality in five of the series (15.6%) (Fig. 1). There was however no evidence that the movement was linked to the slightly longer time our patients spent in the scanner, as movement was shown in these cases to have occurred across the DIXON and standard sequences alike. Those sequences effected by patient motion were adjudged as ‘usual’ in terms of the expected rate in the department by all reviewers, and did not affect anatomical resolution, image interpretability or diagnostic confidence.

**Figure 2:**
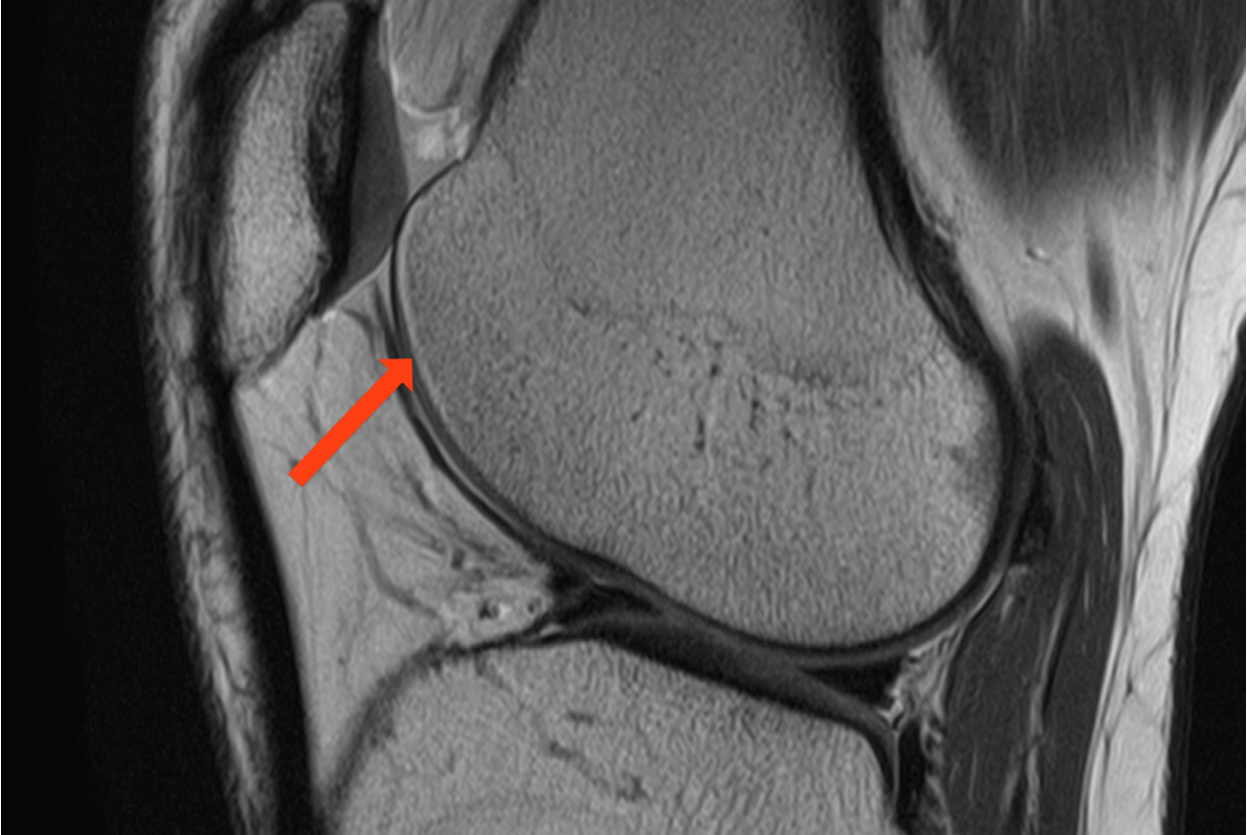
Chemical Shift. The arrow points to the smooth chemical shift present on the DIXON image.

**Figure 3:**
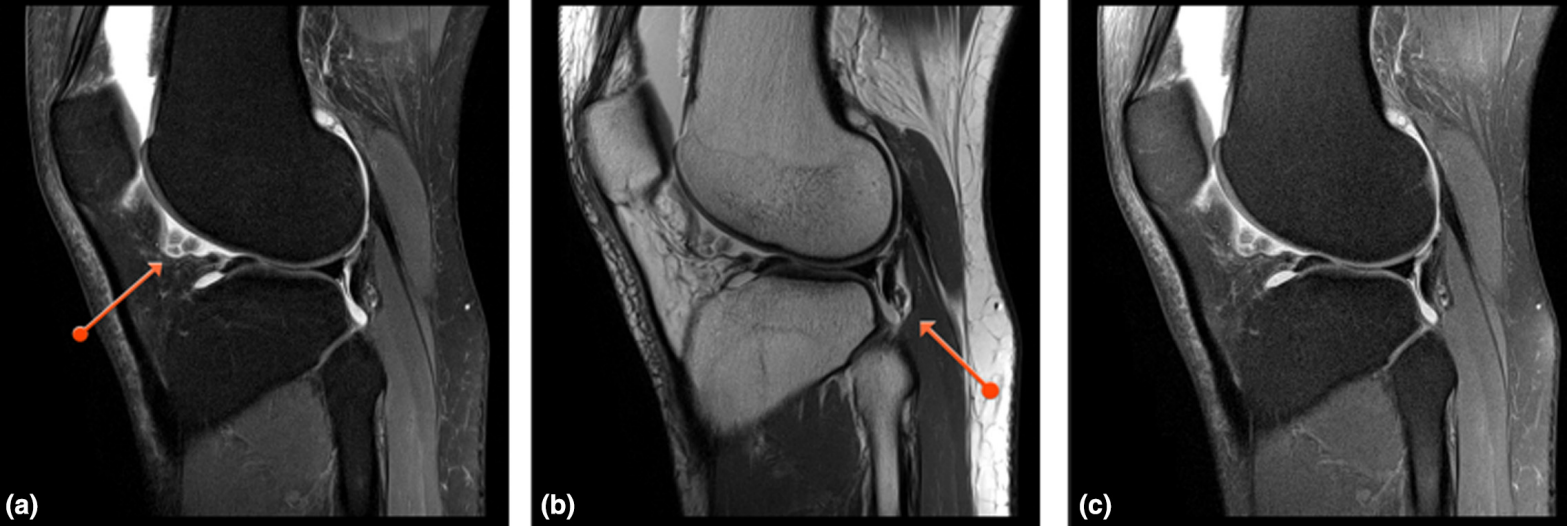
Anatomy. Images (a) and (b) are the DIXON images. Note the clarity around the edges of the cartilage, menisci and sub chondral plates (red arrows) compared with standard PD FS image (c).

**Figure 4:**
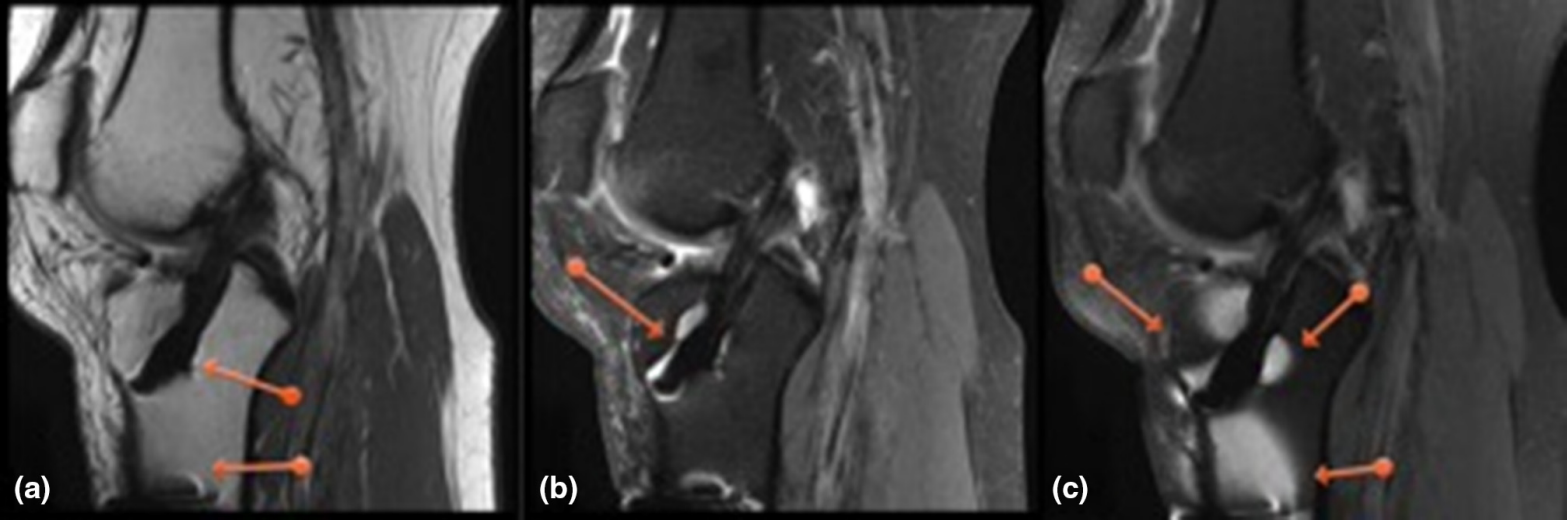
Metal. Images (a) and (b) are the DIXON sequence and c is the standard sequence. The red arrows point to where the chemical saturation (regular FatSat) failed near the metal implants. The DIXON fat saturation failure however was well contained and did not spread beyond the local region. This improvement in robustness around metallic edges is an important advantage of DIXON.

Chemical shift artefact in the DIXON series was present in most cases at the anterior edge of the femoral cartilage, with the effect of making it appear slightly thicker and more obvious than the in the standard PD sequences (Fig. 2). This artefact however was not mentioned by reviewers 2 and 3 and did not affect the ability to assess for cartilage pathology including looking for subtle osseous and soft tissue changes (Fig. 3). The subchondral plate was confidently identified in all cases. The patient who had the metallic implant tolerated the procedure well, and the images were considered diagnostic with minimal artefact degradation (Fig. 4) and the artefact well contained to the local region in the DIXON‐based sequences.

The reviewers were also asked how challenging the image series presented was to report in light of their experience (easy, the usual or difficult). On 15 occasions reviewer 1 stated that the cases were easy, while on only 4 occasions reviewers 2 and 3 thought the case was anything but the usual in terms of read difficulty. Interestingly the only ‘difficult’ rating was given by the first reviewer in a case with a comment on the chemical shift artefact seen. Reviewers 2 and 3 both classified the case as ‘the usual’. There were no real differences in the pathology identified by all reviewers across all cases.

Differences in scoring between the reviewer 1 and reviewers 2 and 3 comes down to the ability to compare with what was taken as the standard acquisition for a knee. Reviewers 2 and 3 had no statistical difference in their scores. These two as a group compared with reviewer 1 had a similar distribution of scoring but on average higher which was felt due to the first reviewer being unblinded and making a direct comparison verses a comparison to usual quality.

The time saving per case in our study was shown to be small (3 min:41 sec vs. 2 min:40 sec + 1 min:30 sec = 4 min:10 sec) along with one less pre‐scan required using the DIXON sequence (estimated time saving 30 sec). The total of approximately 1 min (29 + 30 sec) saved per patient extrapolated across the entire cohort however was approximately 30 min.

## Discussion

**Figure 5:**
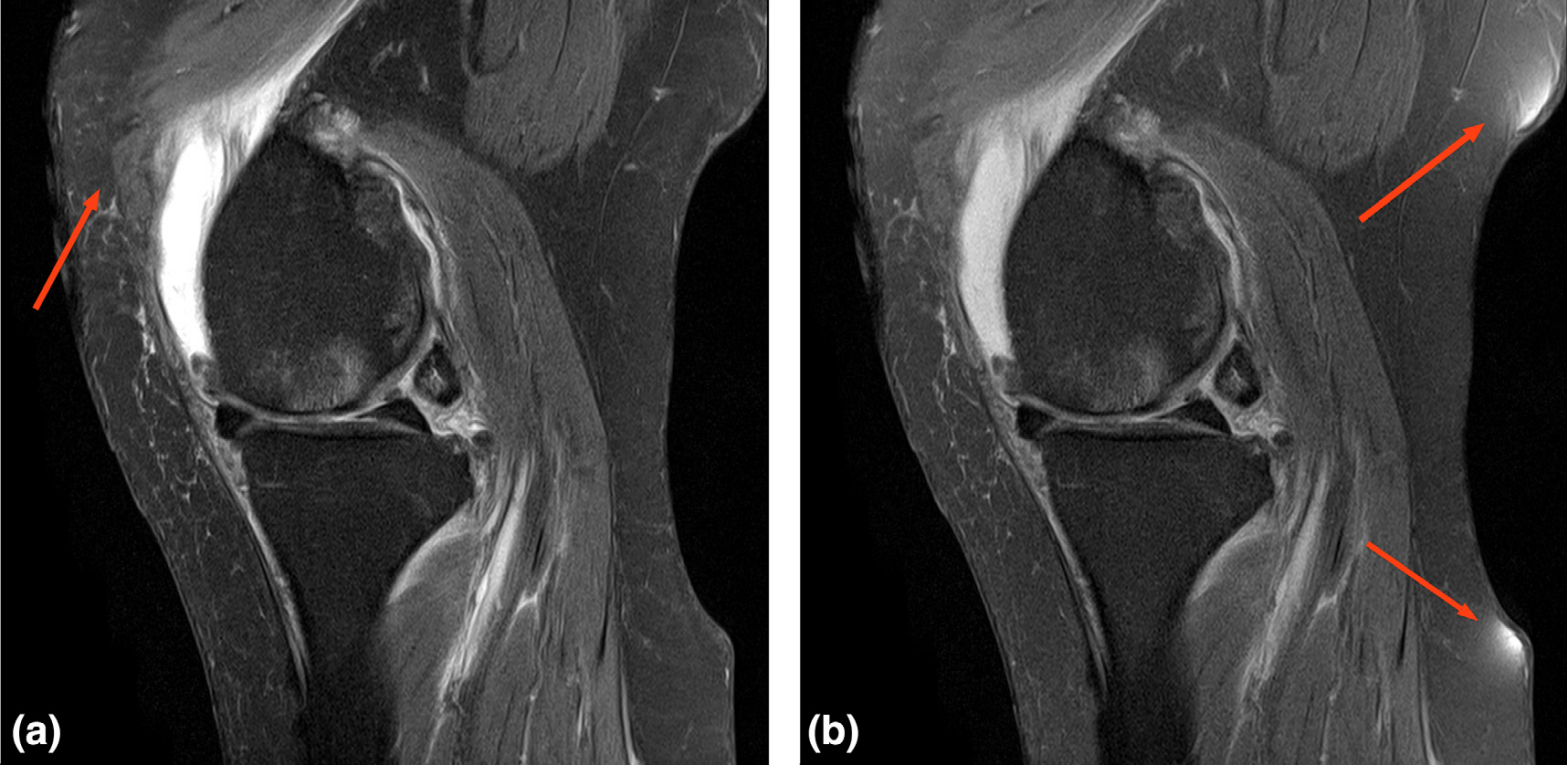
SNR. Note the increased signal to noise ratio broadly in both osseous and soft tissues of the knee in the DIXON sequence (a) verses the conventional sequence (b), and the incomplete fat saturation in (b).

The results of our study have demonstrated that the DIXON sequence is comparable with the standard PD and FS PD sequences. There was no loss in diagnostic confidence for any of the patients for which we conducted this quality survey. Our primary reviewer who had access to all sequences reported diagnostically superior fat saturation across the majority cases, unsurprisingly as DIXON techniques usually produce a higher signal to noise than standard sequences^6^ (Fig. 5).

The time saved per patient was modest at less than a minute, but the advantage conferred by the improved fat saturation and the economy of scale if this time saving was extrapolated out over a month or a year was assessed by our group to be significant. We also believe that as this was a preliminary study into the application of this DIXON sequence, there may be an opportunity for additional sequence parameter adjustments to further shorten acquisition times.

As radiology departments attempt to scan more patients in less time, sequence optimisation becomes critical, and DIXON‐based approaches are potentially very useful in this regard. In the 2016 paper by Park et al,^7^ a comparison of signal to noise resolution (SNR) and contrast to noise resolution (CNR), image sharpness, fat suppression and soft tissue (cartilage/ligament/menisci) interrogation between standard SE acquisitions using a 3‐point Dixon (mDIXON) method was undertaken. The authors found no significant difference in sensitivity, specificity and accuracy between the DIXON sequence and standard T2 SE sequences in regard to diagnostic performance. Additional benefits noted in that study included a decrease in metal artefact, along with a significant time saving of 27% (3 min:40 sec vs. 5 min:00 sec). Our study only managed to save just over 10% in total scan time, though we came into this audit with a routine scan time already significantly lower than the aforementioned study.

Ozgen et al.^8^ utilised the DIXON technique in identification of findings of acute sacroilitis with a multipoint T2 DIXON in comparison to standard T1/T2FS and post‐contrast sequences. They were able to achieve higher CNR for sclerosis and fatty deposition in chronic sacroilitis utilising the fat and water only images. In many cases the DIXON information was more accurate than standard sequences with post‐gadolinium sequences. The ability to assess inflammation, joint space narrowing, chondral changes and erosions requires high‐quality imaging to identify anatomy and inflammation. This DIXON sequence achieves this and thus lends itself to further use in other joints such as the knee.

The paper by Low et al.^9^ assessing the spine by comparing a DIXON method with standard T2 FS SE and STIR imaging also noted a significant scan time savings and equivalent fat suppression and movement artefact. This once again demonstrates the utility of DIXON in different joint and body parts and its ability to demonstrate anatomy and inflammation comparable with standard sequences with a time‐saving benefit (approaching 56%).

All of the aforementioned studies report good diagnostic results; however, none of them make mention of the presence of increased movement artefact and it is therefore difficult for us to say whether this cohort was reflective of the usual incidence of movement in imaging of the knee in our practice. Further investigation is required with a larger sample size. Our findings however indicate that the degree of movement artefact we saw was not detrimental to interpretability.

Overall our study was affected by the artefacts of movement and chemical shift (Fig. 5), albeit minimally, and they did not adversely affect overall image quality, interpretability or diagnostic confidence. Our primary reviewer observed that there was chemical shift artefact in the DIXON sequences though the other reviewers who did not have access to the conventional comparison images did not make this observation. This is presumably due to the smooth nature of this artefact along the cartilage and the fact that it did not impact on interpretability.

The use of the DIXON technique also potentially lends itself to 3D acquisitions and the possibility of a single acquisition with all 4 DIXON elements together with multiplanar reconstruction functionality. Madhuranthakan et al. demonstrated a 3D T2 DIXON acquisition is possible in under 8 min.^10^ This finding suggests a clinically acceptable scan time with the ability for multiplanar interrogation, although further large‐scale studies on image quality are still required.

There were limitations in our study. As mentioned, it was a single institutional review, and a relatively small number of patients were evaluated. Also, though the study was blinded, the two additional reviewers (reviewers 2 and 3) to our MSK expert (reviewer 1) were not blinded to the type of sequences being analysed, and so there is a chance of some bias being introduced. There was also no intra‐observer agreement analysis undertaken in this limited quality audit, and it may have been useful to do this to set a baseline for scoring. In addition, although we have described no significant loss in image quality, interpretability or confidence reviewers 2 and 3 still had standard coronal and axial PD FS sequences with which to review their findings, which may have affected confidence. As a general rule however, this would not have affected the standard search pattern of reviewers 2 and 3. Reviewer 1 had all sequences from which to assess the examination, which allowed a qualitative review side by side of the marrow, cartilage, menisci and ligaments. This was therefore an essentially unblinded study despite the acquisition data being hidden from all reviewers. This was however a prospective study, and we included all patients for whom the routine MRI knee protocol was applicable.

## Conclusions

This study was constructed as a quality audit of our institution’s MRI knee protocol and as part of the wider review of all sequences used to evaluate our patients. The function of the study was to inform the applicability of the DIXON sequence into our routine knee protocol, both in terms of the time the patient spent on the scanner and the diagnostic attributes of the scan series.

We have demonstrated a decrease in image acquisition time that likely improves significantly if optimised and analysed over a monthly or yearly scale.

In addition, the DIXON technique has inherently superior fat saturation due to insensitivities to Bo/B1 field inhomogeneities, especially around metal hardware, making it an ideal sequence in the post‐operative knee.

Further large‐scale studies into other joints and body area may be warranted to assess further applicability as well as investigate any identifiable and fixable cause of the movement artefact we saw in this study. There is also a potential for sequence optimisation and further time savings in the future.

In conclusion, our study provides clear evidence for the utility of the MRI DIXON sequence to be used as a time‐saving method to evaluate the knee, with no loss in diagnostic confidence.

## Conflict of Interest

None of the authors have a conflict or interest in the preparation of the manuscript.

## References

[1] Dixon WT . Simple proton spectroscopic imaging. Radiology 1984; 153: 189–94. 6089263 10.1148/radiology.153.1.6089263

[2] Maeder Y , Dunet V , Richard R , Becce F , Omoumi P . Bone Marrow Metastases: T2‐weighted Dixon Spin‐Echo Fat Images Can Replace T1‐weighted Spin‐Echo Images. Radiology 2018; 286: 948–59. 29095674 10.1148/radiol.2017170325

[3] Eggers H , Brendel B , Duijndam A , Herigault G . Dual‐echo Dixon imaging with flexible choice of echo times. Magn Reson Med 2011; 65: 96–107. 20860006 10.1002/mrm.22578

[4] Xiang QS . Two‐point water‐fat imaging with partially‐opposed‐phase (POP) acquisition: an asymmetric Dixon method. Magn Reson Med 2006; 56: 572–84. 16894578 10.1002/mrm.20984

[5] Berglund J , Ahlstrom H , Johansson L , Kullberg J . Two‐point dixon method with flexible echo times. Magn Reson Med 2011; 65: 994–1004. 21413063 10.1002/mrm.22679

[6] Guerini H , Omoumi P , Guichoux F , et al. Fat Suppression with Dixon Techniques in Musculoskeletal Magnetic Resonance Imaging: A Pictorial Review. Semin Musculoskelet Radiol 2015; 19: 335–47. 26583362 10.1055/s-0035-1565913

[7] Park HJ , Lee SY , Rho MH , et al. Usefulness of the fast spin‐echo three‐point Dixon (mDixon) image of the knee joint on 3.0‐T MRI: comparison with conventional fast spin‐echo T2 weighted image. Br J Radiol 2016; 89: 1062. 10.1259/bjr.20151074 PMC5258172 27008281

[8] Ozgen A . The Value of the T2‐Weighted Multipoint Dixon Sequence in MRI of Sacroiliac Joints for the Diagnosis of Active and Chronic Sacroiliitis. AJR Am J Roentgenol 2017; 208: 603–8. 28004967 10.2214/AJR.16.16774

[9] Low R , Austin M , Ma J . Fast Spin‐Echo Triple Echo Dixon: Initial Clinical Experience With a Novel Pulse Sequence for Simultaneous Fat‐Supressed and Nonfat‐Supressed T2‐Weighted Spine Magnetic Resonance Imaging. J Mag Reson Imaging 2011; 33: 390–400. 10.1002/jmri.22453 21274981

[10] Madhuranthakam AJ , Yu H , Shimakawa A , et al. T(2)‐weighted 3D fast spin echo imaging with water‐fat separation in a single acquisition. J Magn Reson Imaging 2010; 32: 745–51. 20815077 10.1002/jmri.22282 PMC4240221

